# Resistome expansion in disease-associated human gut microbiomes

**DOI:** 10.1186/s40168-023-01610-1

**Published:** 2023-07-29

**Authors:** Simen Fredriksen, Stef de Warle, Peter van Baarlen, Jos Boekhorst, Jerry M. Wells

**Affiliations:** Host-Microbe Interactomics Group, Animal Sciences Department, Wageningen University & Research, Wageningen, The Netherlands

## Abstract

**Background:**

The resistome, the collection of antibiotic resistance genes (ARGs) in a microbiome, is increasingly recognised as relevant to the development of clinically relevant antibiotic resistance. Many metagenomic studies have reported resistome differences between groups, often in connection with disease and/or antibiotic treatment. However, the consistency of resistome associations with antibiotic- and non-antibiotic–treated diseases has not been established. In this study, we re-analysed human gut microbiome data from 26 case-control studies to assess the link between disease and the resistome.

**Results:**

The human gut resistome is highly variable between individuals both within and between studies, but may also vary significantly between case and control groups even in the absence of large taxonomic differences. We found that for diseases commonly treated with antibiotics, namely cystic fibrosis and diarrhoea, patient microbiomes had significantly elevated ARG abundances compared to controls. Disease-associated resistome expansion was found even when ARG abundance was high in controls, suggesting ongoing and additive ARG acquisition in disease-associated strains. We also found a trend for increased ARG abundance in cases from some studies on diseases that are not treated with antibiotics, such as colorectal cancer.

**Conclusions:**

Diseases commonly treated with antibiotics are associated with expanded gut resistomes, suggesting that historical exposure to antibiotics has exerted considerable selective pressure for ARG acquisition in disease-associated strains.

Video Abstract

**Supplementary Information:**

The online version contains supplementary material available at 10.1186/s40168-023-01610-1.

## Introduction

Antibiotic production and resistance are ancient traits important to competition between bacteria [1]. However, medical antibiotic use has driven an increase in antibiotic resistance (ABR) in human- and livestock-associated bacteria [2], and ABR in pathogenic bacteria has become a major concern for human and veterinary medicine [3]. With a One Health perspective in mind, identifying factors driving the spread of ABR in humans, livestock, and the environment is of great importance [4, 5].

Antibiotic resistance is often based on acquisition of antibiotic resistance genes (ARGs). ARGs can spread rapidly in bacterial populations by horizontal transfer both within and across species boundaries via bacteriophages, plasmids, and transposable genetic elements [6, 7]. The epidemiology and spread of ARGs has mainly been studied in clinically relevant bacteria, but the role of the commensal microbiome in the spread of ARGs is of increasing interest [2]. The microbiome contains a stable reservoir of ARGs, collectively termed the resistome. The genes in this reservoir can be spread via inter- and intra-species horizontal gene transfer, enabling pathogenic strains to rapidly adapt upon infection and antibiotic treatment [8–10].

Studies of human, animal, and environmental microbiomes have revealed differences in the abundance and diversity of ARGs (i.e. the resistome) between sites, groups, and populations, suggesting recent or ongoing selective pressure for antibiotic resistance. While antibiotic use induces positive selection for ARG acquisition, other forces act to reduce ARG carriage. ARGs can impart a fitness cost in the absence of antibiotic exposure, and this is thought to select for loss of resistance after cessation of antibiotic treatment [11, 12]. Strain-level microbiome composition, and thus the resistome, may also be equalised within populations by horizontal microbiota transfer [13–16].

Disease-associated resistomes are of particular interest because of their clinical relevance and impact on choice of antibiotic treatment. If a disease is treated with antibiotics, disease-associated microbiome members that acquire corresponding ARGs have a selective advantage. ARGs can co-occur with virulence genes on genomic islands [17], and the two classes of genes may confer synergistic selective advantages to disease-associated strains when co-occurring. These processes may lead to increased ARG abundance (resistome expansion) in disease-associated microbiomes. While some studies have theorised this mechanism to explain differences in ARG abundance between groups [18–22], a comprehensive overview of disease-associated resistomes is lacking. It is not known what resistome differences might be expected due to factors other than natural selection from antibiotic exposure. Microbiome studies are known to be at high risk of confounding factors [23, 24], and unbalanced case/control cohort selection may impact on resistome results. Moreover, diseased hosts may be associated with a distinct but non-disease-specific resistome. Host inflammation and oxidative stress may promote phage lysogeny, increase horizontal gene transfer, and indirectly select for disease-associated bacteria rich in ARGs [19, 25–27]. Currently, it is not known whether there is a consistent link between host disease and an expanded resistome. The difference between the number of studies reporting positive and negative associations could reflect publication bias, as positive associations may be more likely to be published.

In this study, we aimed to provide a comprehensive overview of disease-associated resistomes in human gut microbiome studies. We reasoned that, while comparison of all available data in a common analysis cannot rigorously assess the results of individual studies, as only limited metadata are made publicly available, it would provide a unique perspective on overall trends. We re-analysed 26 studies with publicly available metagenomic data from healthy controls and cases with various morbidities. Some on the included studies investigated diarrhoea and cystic fibrosis, which are commonly treated with antibiotics. Other studies investigated diseases that are not commonly treated with antibiotics, and thus not expected to be associated with an expanded resistome.

## Methods

### Study inclusion and data selection

We aimed to include human gut case-control metagenomic shotgun sequencing studies investigating any disease or morbidity with publicly available raw data and metadata. First, we included studies from the curatedMetagenomicData database [28]. We then conducted a systematic literature review to identify additional metagenomic studies on cystic fibrosis and diarrhoea, diseases commonly treated with antibiotics, by searching PubMed for (((microbiota OR microbiome OR metagenomics) (cystic fibrosis) AND (shotgun))) NOT (Review [Publication Type]). Studies indexed by PubMed before 2022–07-22 were included. Studies without publicly available data and metadata or with less than 10 case samples were excluded. A total of 26 case-control studies were included (Table S1).

We reviewed the metadata of all studies to select either the full sample set or a subset of samples appropriate for case-control comparison. For longitudinal studies, we selected a single sample from each participant. As the included longitudinal studies did not provide detailed metadata on disease symptoms at each timepoint, we opted to use the first sample collected from each participant. For studies that collected samples from multiple cohorts and/or different countries, we split the dataset for separate analysis or excluded samples causing unbalanced study designs. For instance, if the majority of samples were collected in one country and additional cases but no controls were collected in a second country, we excluded these cases. Where possible, we excluded samples from participants who had recently been treated with antibiotics, as we aimed to study evolutionary adaptation of the microbiome to historical antibiotic exposure, rather than the direct effects of ongoing treatment. We included one study on cystic fibrosis despite many cases taking antibiotics, as this is unavoidable and common practice to prevent lung infections [29]. We divided studies into separate datasets when several different diagnoses were investigated and when samples were collected from separate cohorts and/or differed in geography or methodology.

Many datasets did not include all the metadata needed for reproducing the original study results or for novel analysis as was done in this study. An important limitation was that some studies did not provide information on exclusion criteria and recent antibiotic treatment of each participant. We opted not to exclude these datasets because publicly available datasets with complete metadata are so rare that this study would not have been feasible otherwise. The lack of metadata on antibiotic exposure was mainly a problem for studies where antibiotic use was not related to the studied disease, and we note that the datasets in question had neutral resistome case-control differences.

### Data processing

We used NCBI fastq-dump to download all reads from the included samples. We used Kraken 2 [30] for taxonomic assignment of reads. The Kraken database was compiled on 2022-08-15 and included all default taxonomy options (i.e. prokaryote, plasmid, viral, fungal, protozoan, human, and plant genomes) and additionally four roundworm and flatworm genomes (GCA_003958945.1, GCA_900618425.1, GCA_000941615.1, and GCA_000936265.1). To accurately assess the relative abundance of the resistome, we normalised for the proportion of reads classified as any other domain than bacteria (otherwise, a sample with 50% host reads would appear to have half the ARG abundance of an equivalent sample with no host DNA contamination). This adjustment had a little impact on most studies, as the proportion of non-bacterial reads was low (Fig. S1).

We used MMseqs2 [31] alignment to the ResFinder database [32] to identify ARGs and quantify their abundance. To reduce noise from ambiguous mapping to highly similar gene variants, we clustered all sequences to 90% identity using MMseqs2 easy-cluster with settings ‘-min-seq-id 0.9 -cov-mode 0’. We mapped metagenomic reads to the representative sequences of the clusters using MMseqs2 easy-search with setting -s 4.500 and accepted the best hit with minimum 50 bp alignment and 80% identity. ARG abundance was normalised to reads per kilobase per million reads (RPKM).

### Statistical analysis

To determine whether the case and control groups of each study differed in total ARG abundance, we summed the RPKM abundance of all ARGs in each sample and calculated the mean per group. We used unpaired Wilcoxon rank-sum test to assess statistical significance. We used R v4.2.3 package vegan v2.6.4 [33] function RDA for principal component analysis (PCA) and redundancy analysis (RDA), function vegdist to calculate Bray–Curtis dissimilarity, and function adonis to perform PERMANOVA to determine the overall compositional difference. When estimating mean genome sizes with MicrobeCensus v1.1.0 [34], we used the default settings and included only samples with < 5% eukaryotic DNA abundance.

## Results and discussion

### Cases with antibiotic-treated diseases feature expanded resistomes

**Fig. 1:**
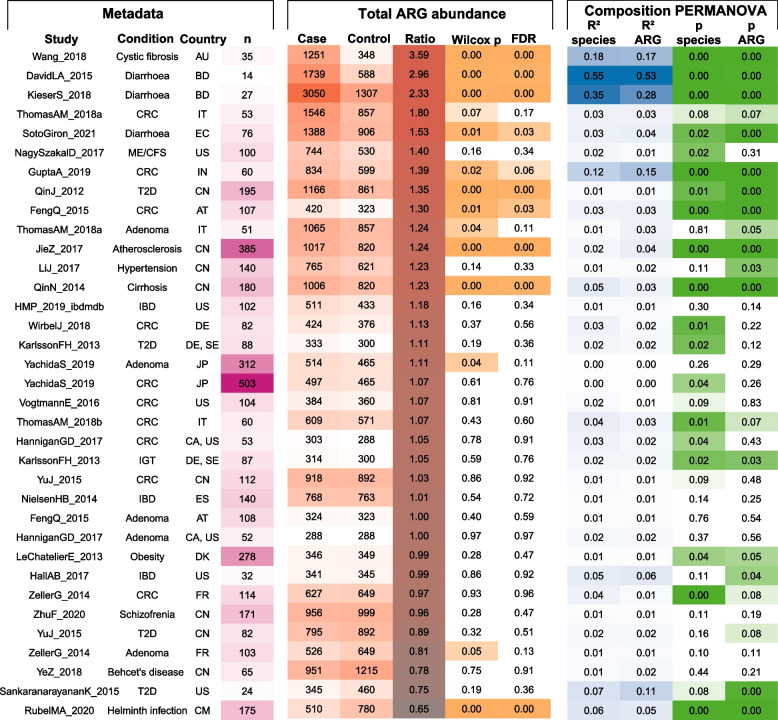
Resistome case-control associations. Summary statistics per study/disease, sorted from strongest to weakest total ARG abundance case association. The columns under total ARG abundance show mean reads per kilobase per million reads (RPKM) total ARG abundance in case and control samples, the ratio of these, and Wilcoxon rank-sum test *p* value for case vs control samples per study. The PERMANOVA columns show Bray–Curtis dissimilarity PERMANOVA *R*^2^ and *p* values for species-level taxonomy and ARG composition. The PERMANOVA model only compared cases to controls and did not account for any potential confounding variables due to the limited availability or completeness of such metadata. CRC, colorectal cancer; adenoma, non-cancerous tumour; ME/CFS, chronic fatigue syndrome; T2D, type 2 diabetes; IGT, impaired glucose tolerance; IBD, inflammatory bowel disease

**Fig. 2:**
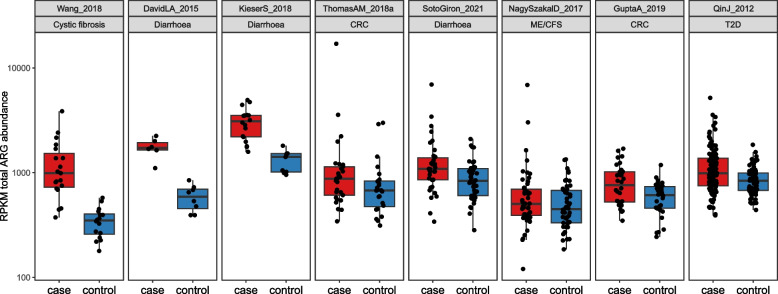
Boxplot of the total ARG abundance for the four datasets with the strongest case association in ARG abundance. Boxplots for all datasets are shown in Fig. S2

Case-control studies on cystic fibrosis and diarrhoea, diseases for which antibiotics are the main treatment, showed greater disease-associated resistome expansion (higher ARG abundance in cases than in healthy controls within the dataset) than studies on diseases not treated with antibiotics (*p* = 0.0001, Wilcoxon rank-sum test). The four datasets of antibiotic-treated diseases were all among the five studies with the greatest resistome expansion in cases. Of the 35 datasets, eight had significantly (*p* < 0.05, Wilcoxon rank-sum test with FDR correction) higher total ARG abundance in cases while only one study had significantly lower ARG abundance in cases (Fig. 1). While total ARG abundance was highly variable between individuals, case and control samples had limited overlap in the studies with the greatest case-associated resistome expansion (Fig. 2). We list case-control comparison for individual ARGs within each study in Table S2 and species-level taxonomic differences in Table S3. Confounding variables were not accounted for in the main analysis because such metadata were only sporadically available. This is a fundamental limitation of re-using publicly available data. Ideally, we would have consistently accounted for resistome associations with factors such as age [35, 36], sex [37], diet [38, 39], and exposure to livestock [40]. We note that age, the most commonly available variable, often correlates with total ARG abundance (Fig. S1), but that this effect is variable and cannot explain large case-control differences.

The largest difference in ARG abundance between case and control participants was found in cystic fibrosis (CF) patients, who had on average 3.59 times the ARG abundance of healthy controls (*p* = 0.000003, Wilcoxon rank-sum test with FDR correction). Although a number of studies have investigated the CF-associated microbiome [41], only a single, Australian dataset [42, 43] met our inclusion criteria. While the Australian CF cases had high total ARG abundance compared to the study controls, they were still comparable to both case and control samples collected in other studies from countries with higher antibiotic usage, such as Bangladesh and China (Fig. 1). It is possible that horizontal microbiota transfer between individuals within the Australian population, where antibiotic use and ABR levels are low, limits ARG abundance in patients by continuously introducing susceptible strains. CF patients in countries with high baseline ARG abundance in the general population likely reach higher ARG abundances.

### The diarrhoea-associated resistome

**Fig. 3:**
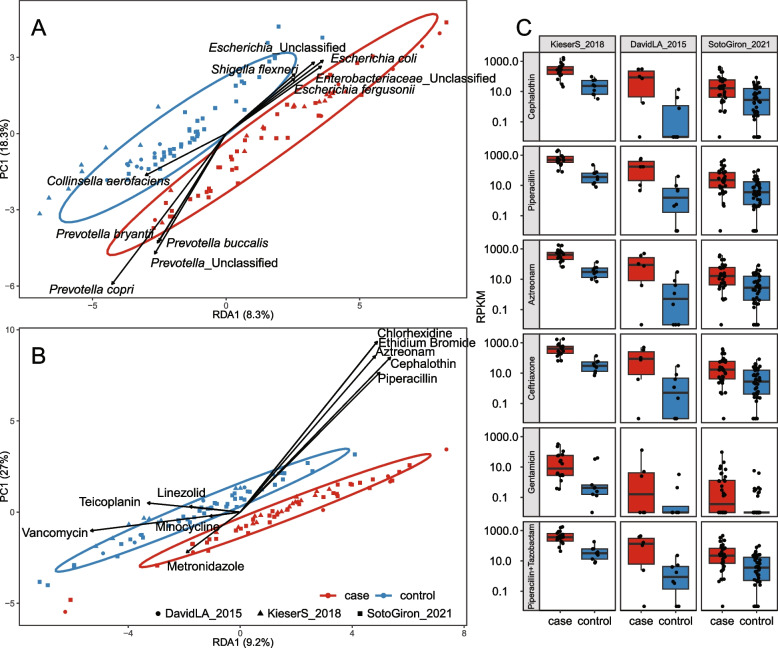
The diarrhoea-associated microbiome and resistome. **A** Redundancy analysis (RDA) showing the species most strongly separating case and control samples in diarrhoea studies (on axis RDA1). Each label represents a single sample, ellipses represent 95% confidence level, and arrows indicate taxa driving the sample separation; samples in the direction the arrow is pointing have a higher abundance of the taxon. Species relative abundance input data were transformed by log(1000 × abundance + 1), and the study was used as RDA covariate. **B** RDA on the summed abundance of all ARGs per ResFinder (conferred) resistance phenotype. **C** Boxplots of the main ResFinder ARG phenotypes separating case samples on the RDA axis, excluding disinfectant resistance genes. Corresponding analysis at the ARG level is shown in Fig. S5

The diarrhoea-associated microbiome has been reported to have a distinct compositional profile influenced by exposure to antibiotics [20, 21, 44–47]. Our re-analysis of three publicly available diarrhoea datasets found that although the causative agent may vary, diarrhoea cases share an increased abundance of *Enterobacteriaceae* species including *Escherichia coli*, *Salmonella enterica*, *Shigella dysenteriae*, and *Klebsiella pneumoniae* (Fig. 3A). The occurrence and abundance of *Vibrio cholerae* was limited, except for in the dataset from David et al. [44], which specifically studied *Vibrio cholerae*-associated diarrhoea.

Despite excluding samples collected after antibiotic treatment (to avoid confounding evolutionary adaptation of the microbiome to historical antibiotic exposure and direct effects of ongoing treatment), we found strong expansion of the diarrhoea-associated resistome. This suggests that bacterial strains that are more prevalent and/or abundant in the gut microbiome of diarrhoea cases have adapted to frequent exposure to antibiotic treatment. The dataset from Soto-Girón et al. [47], which assessed both urban and rural diarrhoea cases in Ecuador, showed a 53% increase in total ARG abundance in cases. The datasets from Kieser et al*.* [21] and David et al*.* [44], both using samples collected in Bangladesh, showed double and triple increase in total ARG abundance in cases. In both studies, case and control participant groups were not entirely equivalent. In Kieser et al. [21], there was a mismatch in age and social class, and David et al. [44] included two cohorts sampled at different times, with only cohort 1 including healthy controls and only cohort 2 including cases sampled prior to antibiotic treatment. In all three studies, the cases had a high abundance of ARGs conferring resistance to cephalothin, piperacillin, aztreonam, spiramycin, ceftriaxone, and gentamicin (Fig. 3B–C). The most significant case-associated ARG phenotypes per study were ampicillin resistance in Kieser et al. [21] (655 vs 68 RPKM, *p* = 0.00002), cefoxitin in Soto-Girón et al. [47] (127 vs 62 RPKM, *p* = 0.002), and chloramphenicol in David et al. [44] (104 vs 6 RPKM, *p* = 0.02). Single genes such as *bla*_TEM_ (conferring resistance to beta-lactams) contributed up to 20% of the total increase in ARG abundance in cases within all studies, but case-associated resistome expansion was also to a large extent driven by less abundant genes (Fig. S4).

### Differences in ARG abundance in diseases not treated with antibiotics

In addition to taxonomy and antibiotic exposure, the resistome may differ between case and control groups due to confounding factors. These may for instance include bias in participant selection, or inflammation facilitating colonisation by generalist disease-associated strains enriched in ARGs (i.e. strains associated with several different diseases or the hospital environment). To test whether such factors might drive a general resistome expansion in microbiome study case participants, we included studies on a range of different morbidities [48–70]. Although case-control differences were smaller than in the studies on antibiotic-treated diseases, we observed some differences between studies (Fig. 1).

Inflammatory bowel disease (IBD, including both ulcerative colitis and Crohn’s disease) involves bouts of intestinal inflammation. Early-life antibiotic usage has been suggested to predispose to IBD [71], and antibiotics may in some cases be used to treat IBD complications [72], but antibiotics are not a generally used treatment. All the three re-analysed IBD datasets [50, 52, 58, 73, 74] showed weak overall species-level compositional differences (Fig. 1), with some individual low-abundance species such as *Akkermansia muciniphila*, *Fusobacterium nucleatum*, and various *Alistipes* species differing in abundance between cases and controls. We found high inter-individual variation and different results between studies, and a caveat to this analysis was that we lacked metadata on the symptom severity, a key variable in determining the IBD-associated microbiome. Although we cannot examine the datasets as rigorously as the original authors, we note that none of the three re-analysed IBD datasets showed resistome case-control associations. Neither the overall resistome nor individual ARGs with abundances greater than 0.1 RPKM differed significantly between cases and controls.

**Fig. 4:**
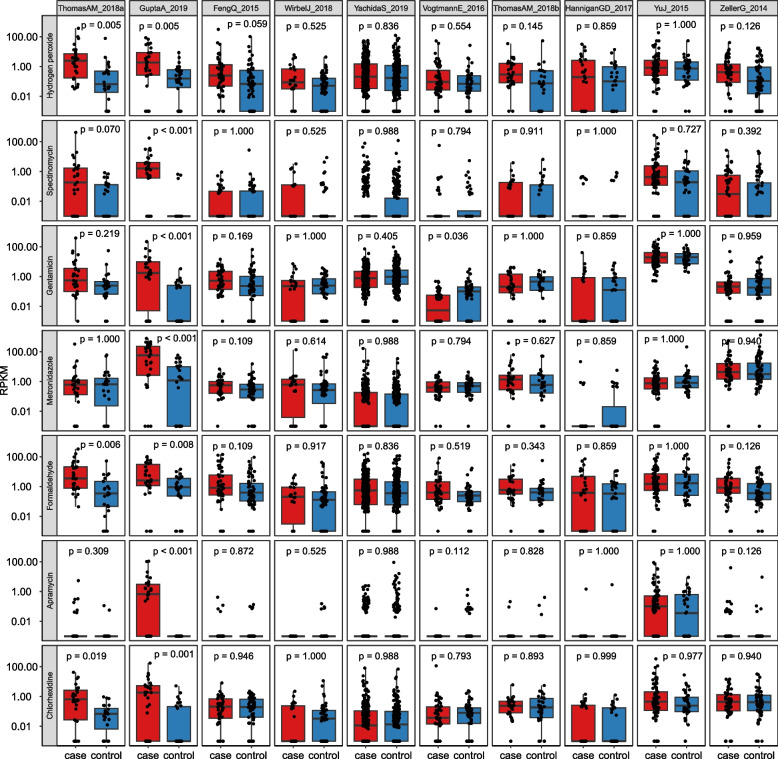
Boxplot of the ResFinder ARG phenotypes that showed the strongest case associations in the three CRC studies (excluding adenoma samples) with strong overall resistome expansion in cases (ratio > 1.2). Datasets (columns) are sorted from left to right by the strongest to weakest total ARG abundance case association. ResFinder ARG phenotypes (rows) are sorted from strongest (top) to weaker (bottom) case association in RDA analysis constrained by case-control status with study as covariate. *p* values are calculated by Wilcoxon rank-sum test and FDR corrected within each study for the number of ResFinder phenotype categories compared

Several studies have investigated the microbiome associated with colorectal cancer (CRC) [48, 49, 51, 63–66, 68, 69]. CRC is not treated with antibiotics, so the CRC-associated microbiome should not face increased selection pressure for ARG expansion. However, the CRC case-associated resistome might be influenced by confounding factors such as lifestyle and diet that predispose to CRC development in individuals with a CRC risk genotype [75]. Most CRC datasets showed no significant difference in total or individual ARG abundance between cases and controls. However, Gupta et al. (India) [49], Feng et al. (Austria) [48], and Thomas et al. cohort 1 (Italy) [63] trended towards higher total ARG abundance in cases (Fig. 1). This was driven by different ARGs in each study (Fig. 4, Table S2), but ARGs conferring resistance to disinfectants played a role in the three studies with the strongest case-associated resistome expansion.

### Confounding variables and unbalanced study designs

Ideally, a case-control study design should involve case and control populations identical in all aspects except for the investigated disease. Such designs are feasible in laboratory experiments, but research on humans present large individual variation and lifestyle-related environmental differences [23, 24]. This poses challenges because the microbiome and risk of disease development may be independently correlated not only with commonly recorded factors but also with factors difficult to record or quantify, such as dietary habits [76] and socioeconomic status [77, 78]. Case participants may also acquire hospital-associated strains rich in ARGs through horizontal transfer.

We found that different studies on the same disease may find different resistome case-control associations, despite species-level taxonomic change being in agreement. Studies investigating different CRC cohorts showed (trends towards) both higher and lower ARG abundance in cases, despite seemingly equivalent participant selection criteria. In addition to variation between studies, the two separate Italian CRC cohorts reported in Thomas et al. [63] differed. Cohort 1/A (collected in Vercelli) was characterised by high overall ARG abundance, which was expanded in cases compared to controls. ARG abundance in cohort 2/B (collected in Milan) was lower and similar in cases and controls. Several individual ARGs were significantly differentially abundant in cohort 1, but no ARGs were significantly different within cohort 2. A previous meta-analysis found both cohorts to have a CRC-associated taxonomic compositional profile in agreement with other CRC studies [63, 65]. Gupta et al. collected cases and controls from the same locations, but controls were collected as part of a separate study, and this may have contributed to greater differences in taxonomy and resistome compared to the other CRC studies. All microbiome studies use inclusion criteria to limit the impact of confounding variables on results, but it is possible that the interpretation and application of sampling criteria vary within and between studies. Strict participant selection criteria and collection of additional metadata are warranted in human resistome studies.

### Linking taxonomic composition and the resistome

In order to assess the immediate clinical relevance of an ARG, it is key to determine its host and genomic context. Unfortunately, short-read metagenomic sequencing data is not well suited for assessing the genomic context of ARGs [10, 79–81]. Identical ARG copies occur in different taxa due to horizontal gene transfer, and adjacent genomic regions may also be shared if included in a mobile genetic element or genomic island. Among the re-analysed datasets, some studies showed large differences in both taxonomic composition and ARG abundance. In other studies, such as the Jie et al. (atherosclerosis) and Qin et al. (type 2 diabetes), ARG abundance was 20–40% higher in cases despite case/control status only explaining 1–3% of the species level dataset variation (Fig. 1). Differences in the resistome and taxonomic composition may occur due to interdependencies (e.g. colonisation by different species inherently carrying different ARGs) or independently (abundance shifts among closely related strains with variable ARG content), but these scenarios cannot be differentiated by short-read metagenomic sequencing. This makes it difficult to determine whether increased ARG abundance occurs due to conferring an ecologically relevant trait in disease-associated strains or by correlation with taxonomic change. Taxa may differ in ARG content due to intrinsic resistance and variable selective pressures exerted by each antibiotic, and different ARGs may confer the same trait. Furthermore, ARGs are often located on plasmids as a part of the accessory genome and variably present in closely related lineages [82, 83]. This allows resistomes to differ despite limited observed changes in taxonomic composition.

Furthermore, while some ARGs may have strong correlations linking them to specific species [84], this is rarely the case for the ARGs driving case-associated resistome expansion. In the datasets with resistome expansion in cases, we found strong (auto) correlation in the case-control association of ARGs and the species with which they had the strongest correlation with, despite weak sample-to-sample correlations (Fig. S6). Ultimately, it is very challenging to connect the presence and abundance of individual genes with each other and overall taxonomic abundance using short-read sequencing data. Recent work has successfully utilised long-read [85, 86] and Hi-C [87–91] sequencing, and future studies aiming to study the resistome may benefit from implementing these strategies.

Resistome studies may also reveal differences in ARG abundance as a technical artefact of metagenomic sequencing methodology. Bacteria may carry (multiple) ARGs on (high-copy-number) plasmids, thus contributing greatly to the observed resistome compared to strains with a single chromosomal copy of the same ARG. Variable genome size may also influence a strain’s contribution to the observed resistome. A microbiome with a high abundance of taxa with small genome sizes but an average number of ARGs will appear to have an abundant resistome. We do not expect this to have contributed meaningfully to the strongest case-control differences reported in the present study, as commonly case-associated *Gammaproteobacteria* species have larger genomes than commonly control-associated *Lactobacillus* and *Prevotella* species. Estimation of the mean genome size per sample within the diarrhoea datasets using MicrobeCensus [34] showed no significant difference in mean genome sizes between case and control samples. Plasmid carriage may play a modest role in expanded case-associated resistomes as *Enterobacteriaceae* commonly carry ARGs on plasmids, although these are large and occur in low copy numbers [92]. Data on plasmid copy numbers in different (commensal) taxa could be of great relevance for resistome research, but we are not aware of any comprehensive work on this topic.

## Conclusions

The human gut resistome is highly variable between individuals, but strong differences can still be observed between groups in case-control studies. Antibiotic treatment of disease appears to exert strong positive selection pressure for acquisition and maintenance of ARGs on the disease-associated microbiome, driving observable expansion of the disease-associated resistome. This implies that the disease-associated microbiomes contain strains with high disease specificity that, while transmitted between individuals and a part of the resident microbiota, are more prevalent and/or abundant during disease and antibiotic treatment. High baseline resistome abundance in controls does not appear to limit further (additive) resistome expansion, underpinning the importance of limiting antibiotic use in populations with high resistance levels. The resistome of case and control groups may also show differences without any clear biological explanation, and future resistome research should take great care in selecting equivalent study cohorts.

## Supplementary Information

**Additional file 1: Table S1. **Table showing the internal study name, PubMed ID, DOI, and title of the re-analysed datasets.

**Additional file 2: Table S2. **Table showing the mean abundance (RPKM) of each ARG in cases and controls within each study.

**Additional file 3: Table S3. **Table showing the mean relative abundance (%) of each species-level taxonomic classification in cases and controls within each study.

**Additional file 4: Table S4. **Key metadata of the included samples. Samples used within several datasets, such as controls from studies on CRC and adenoma, are listed in 2 separate rows.

**Additional file 5: Fig. S1. **Boxplots showing domain-level abundance within each study.

**Additional file 6: Fig. S2. **Scatterplots comparing age and total ARG abundance within each dataset.

**Additional file 7: Fig. S3. **Boxplots comparing the total abundance of all ARGs in case and control samples from all datasets. Some included studies covered multiple different diagnoses; cases with multiple diagnoses and controls corresponding to multiple diagnoses are shown in more than one facet.

**Additional file 8: Fig. S4. **Plots showing the relative contribution of individual ARGs towards overall resistome expansion. While a few ARGs contribute a large proportion of the total ARG abundance expansion, many ARGs also trend towards case-association. Thus, they contribute to overall resistome differences without themselves being significantly different. The ARGs with the strongest impact are labelled.

**Additional file 9: Fig. S5. **ARG-level analysis of the three diarrhoea datasets. A) RDA constrained by case-control status. B) Boxplots showing the abundance of the ARGs with the strongest impact on the RDA1 axis.

**Additional file 10: Fig. S6. **Strong (auto) correlation between the case-association of ARGs and species despite limited sample-by-sample co-occurrence. This figure shows the relationship between the disease-association of ARGs and the species they are most strongly correlated with (regardless of strength and significance of this correlation). Points represent pairs of each ARG (minimum abundance of > 1 RPKM) and the species (minimum abundance of > 0.01%) it has the strongest positive Spearman’s rank correlation coefficient with. High positive values on the x- and y-axis indicate case-association of the ARG and species, respectively. The purple line indicates 1:1 equal case-control association of ARG and species, which could be expected if the ARG is found only on the chromosome of a single species of average genome size. David et. al. 2015 has several, likely genuine, strong correlations due to consistently high abundance of *Vibrio cholerae*in cases.

## Data Availability

No new data was generated for this study. The accession numbers of all analysed samples are available in Supplementary Table S4.
