# Supplementary figures and images for: Resistome expansion in disease-associated human gut microbiomes
Source: Microbiome. 2023 Jul 29;11:166. doi: 10.1186/s40168-023-01610-1 (PMC10386251; doi:10.1186/s40168-023-01610-1)

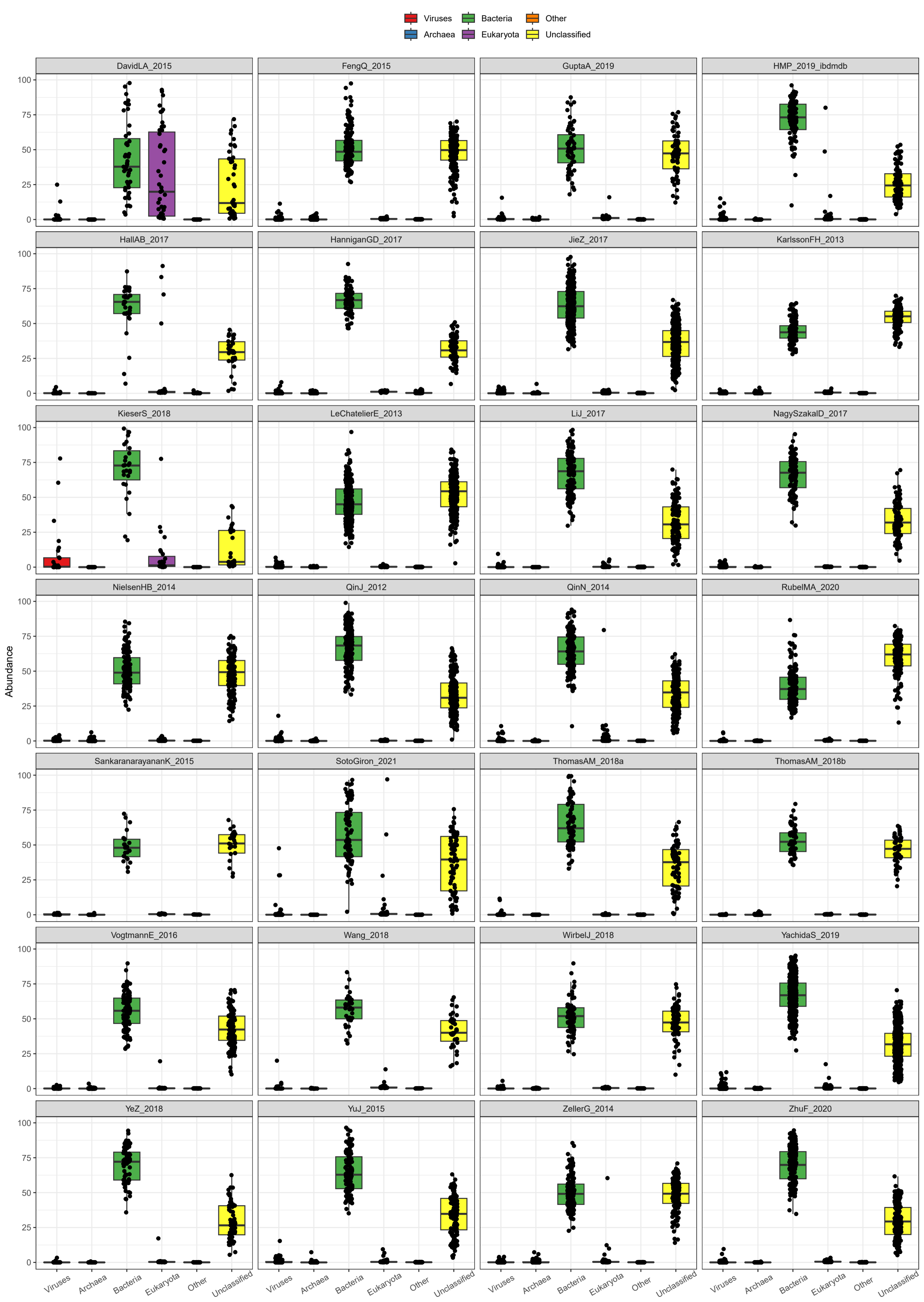

Supplement: Supplementary file 6 — Additional file 5: Fig. S1. Boxplots showing domain-level abundance within each study. [file 40168_2023_1610_MOESM5_ESM.pdf]

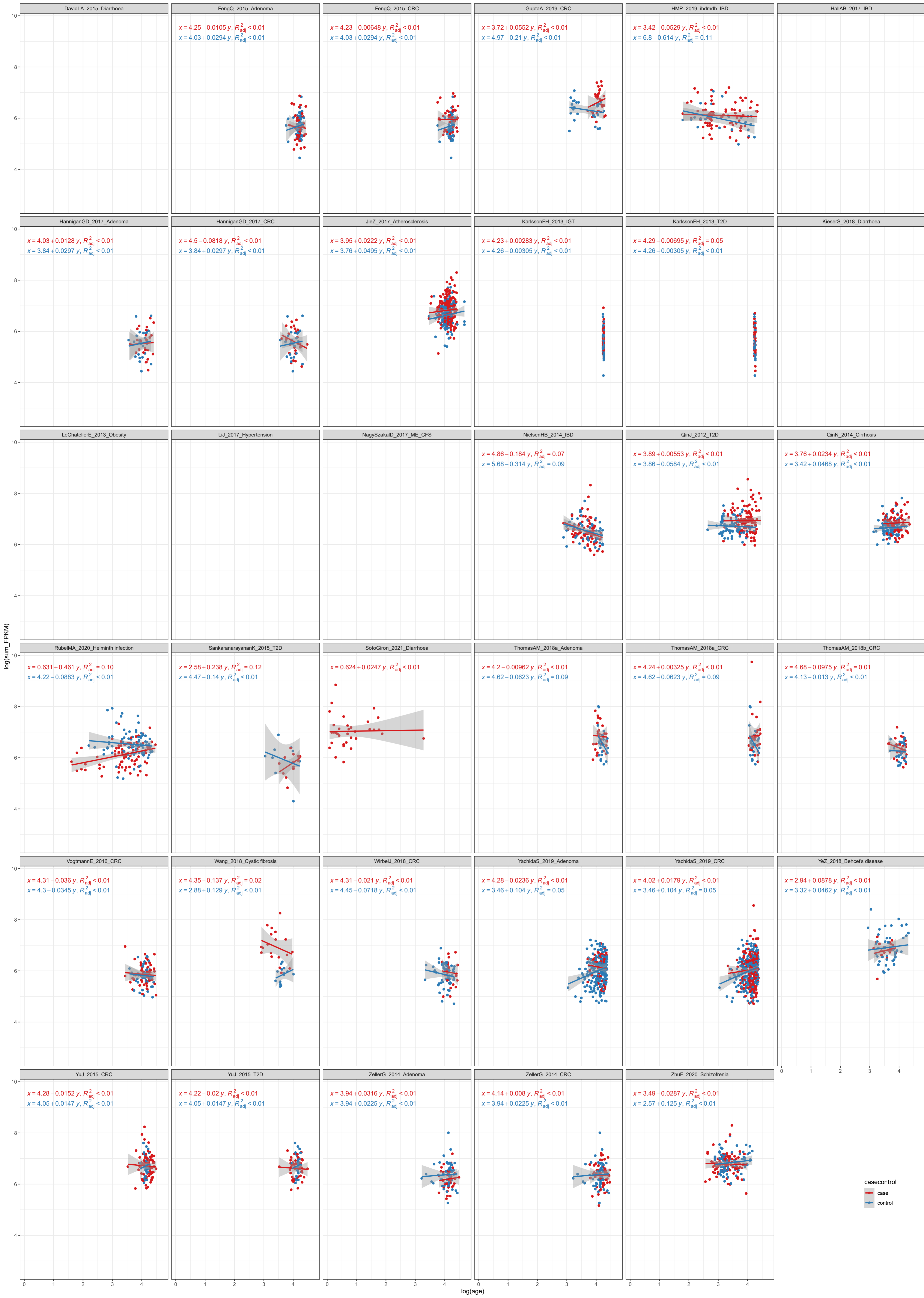

Supplement: Supplementary file 7 — Additional file 6: Fig. S2. Scatterplots comparing age and total ARG abundance within each dataset. [file 40168_2023_1610_MOESM6_ESM.pdf]

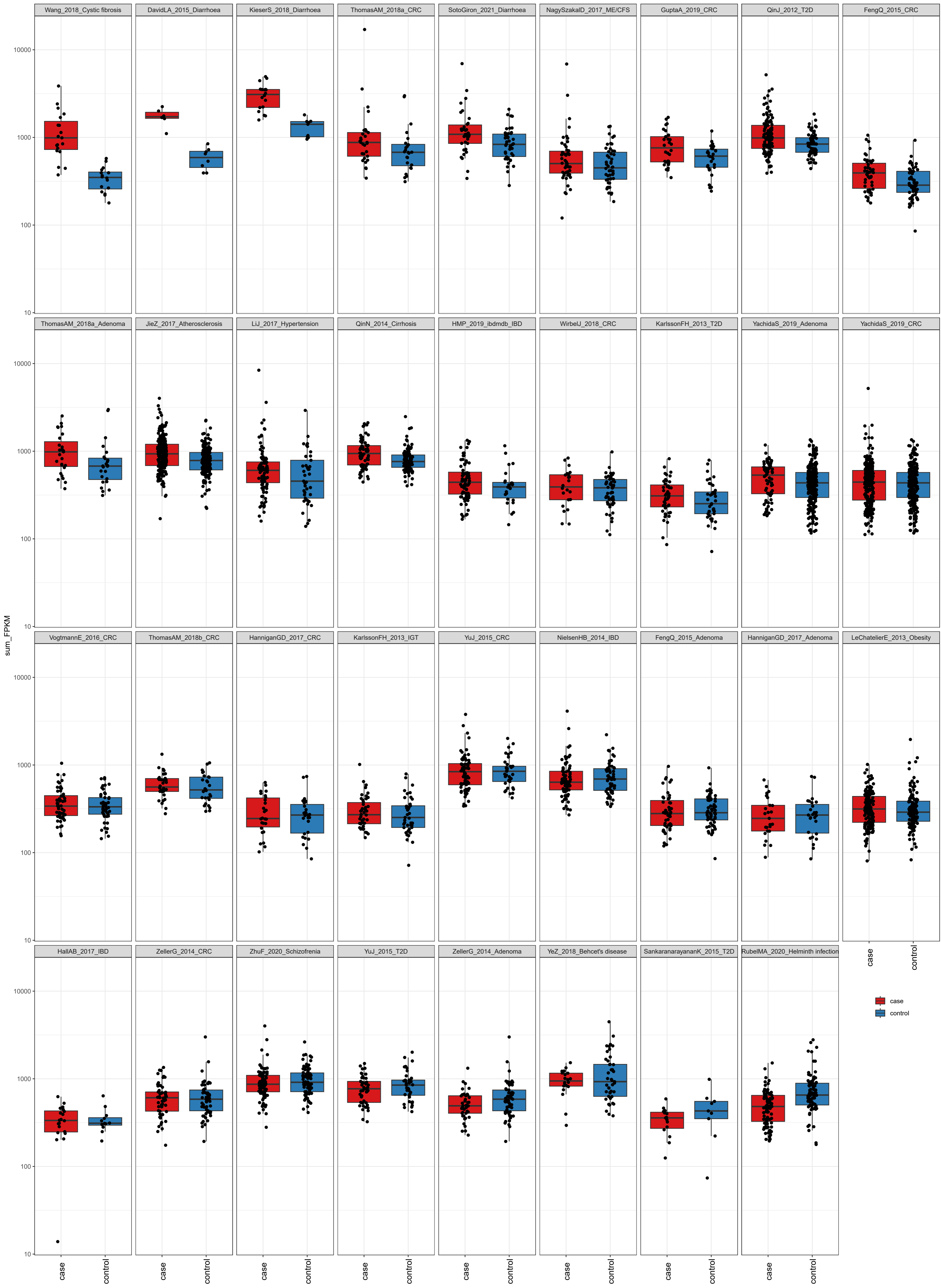

Supplement: Supplementary file 8 — Additional file 7: Fig. S3. Boxplots comparing the total abundance of all ARGs in case and control samples from all datasets. Some included studies covered multiple different diagnoses; cases with multiple diagnoses and controls corresponding to multiple diagnoses are shown in more than one facet. [file 40168_2023_1610_MOESM7_ESM.pdf]

● Case\_associated ● Control\_associated

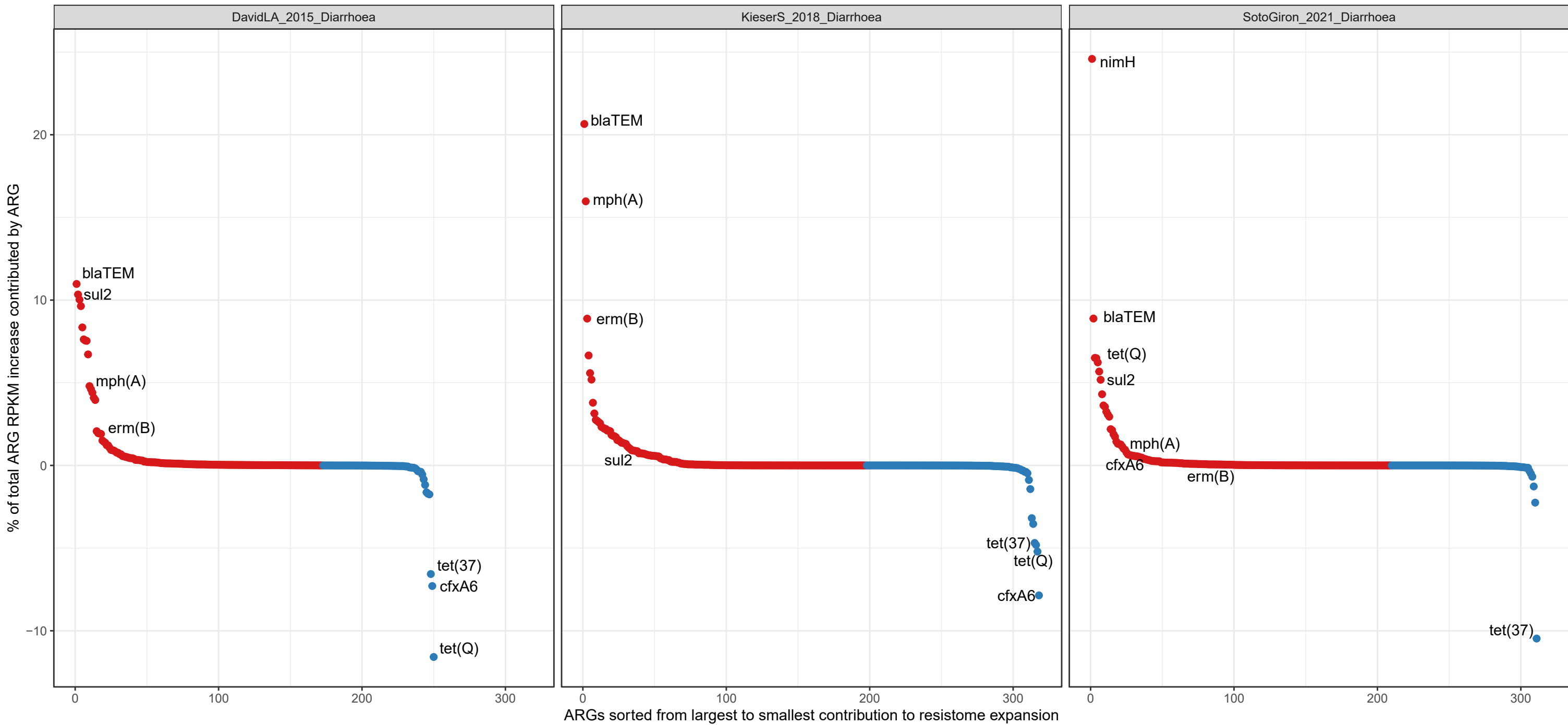

Supplement: Supplementary file 9 — Additional file 8: Fig. S4. Plots showing the relative contribution of individual ARGs towards overall resistome expansion. While a few ARGs contribute a large proportion of the total ARG abundance expansion, many ARGs also trend towards case-association. Thus, they contribute to overall resistome differences without themselves being significantly different. The ARGs with the strongest impact are labelled. [file 40168_2023_1610_MOESM8_ESM.pdf]

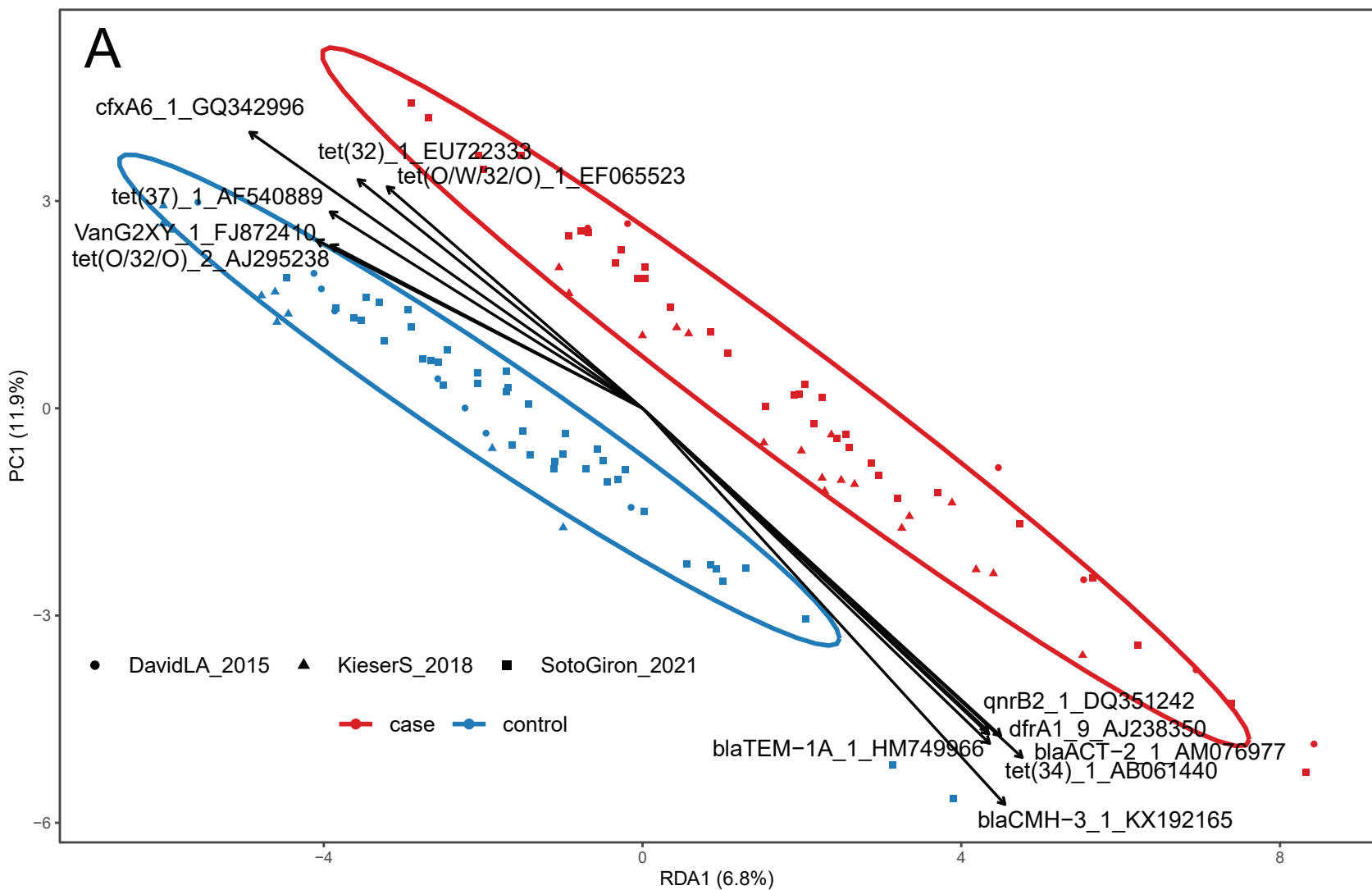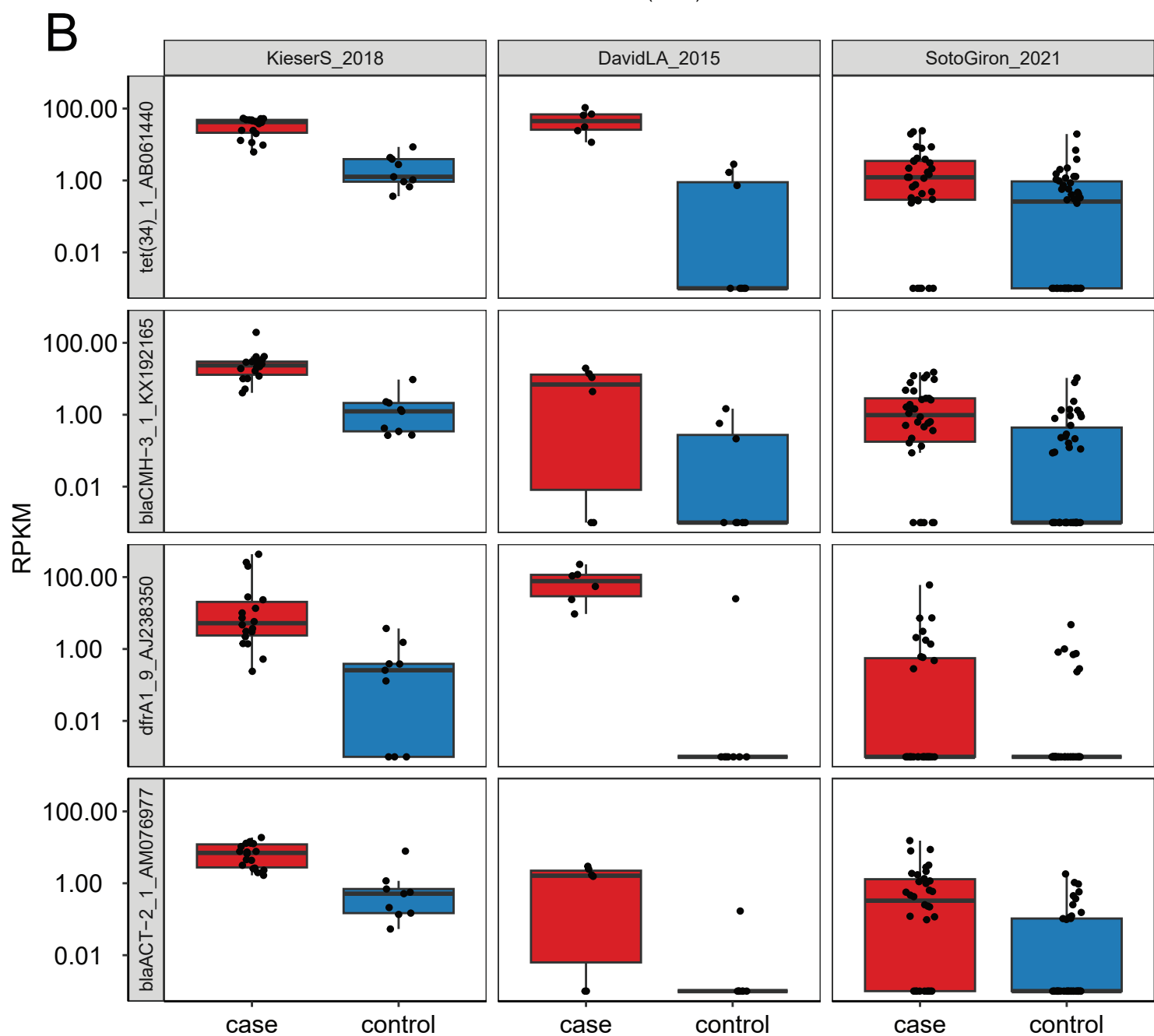

Supplement: Supplementary file 10 — Additional file 9: Fig. S5. ARG-level analysis of the three diarrhoea datasets. A) RDA constrained by case-control status. B) Boxplots showing the abundance of the ARGs with the strongest impact on the RDA1 axis. [file 40168_2023_1610_MOESM9_ESM.pdf]

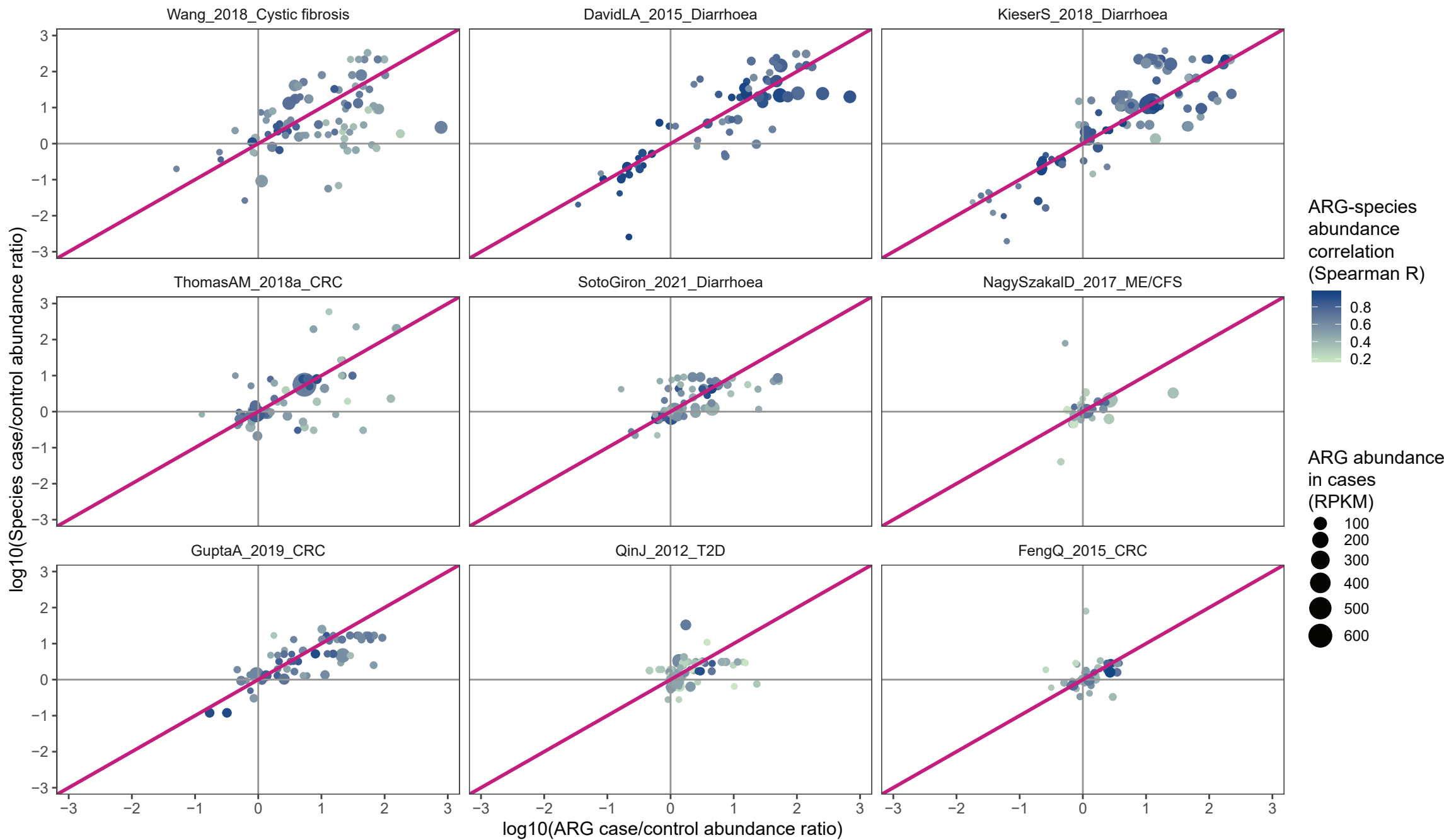

Supplement: Supplementary file 11 — Additional file 10: Fig. S6. Strong (auto) correlation between the case-association of ARGs and species despite limited sample-by-sample co-occurrence. This figure shows the relationship between the disease-association of ARGs and the species they are most strongly correlated with (regardless of strength and significance of this correlation). Points represent pairs of each ARG (minimum abundance of > 1 RPKM) and the species (minimum abundance of > 0.01%) it has the strongest positive Spearman’s rank correlation coefficient with. High positive values on the x- and y-axis indicate case-association of the ARG and species, respectively. The purple line indicates 1:1 equal case-control association of ARG and species, which could be expected if the ARG is found only on the chromosome of a single species of average genome size. David et. al. 2015 has several, likely genuine, strong correlations due to consistently high abundance of Vibrio choleraein cases. [file 40168_2023_1610_MOESM10_ESM.pdf]
